# Glutathione reactivity with aliphatic polyisocyanates

**DOI:** 10.1371/journal.pone.0271471

**Published:** 2022-07-15

**Authors:** Adam V. Wisnewski, Jian Liu

**Affiliations:** Department of Internal Medicine, Yale University School of Medicine, New Haven, Connecticut, United States of America; University of Nebraska Medical Center, UNITED STATES

## Abstract

Isocyanate chemicals known to cause adverse health effects when inhaled are essential to making important products and are used in multiple industries. Glutathione (GSH), a major antioxidant of the lower airways with a well described role in xenobiotic metabolism, is a primary reaction target for di-isocyantes. However, GSHs reactivity with poly-isocyanates which have largely replaced diisocyanates (particularly aliphatic) in most end-user settings remains uncertain. We hypothesized aliphatic polyisocyanates would readily react with glutathione under physiologic conditions and the products could be identified using liquid chromatography (LC) coupled-mass spectrometry (MS) and tandem MS/MS. The data identified (tris)GSH-isocyanate adducts as the major reaction product of GSH with the most commonly used contemporary polymeric (tri-isocyanate) formulations of hexamethylene diisocyanate (HDI), the isocyanurate and biuret, as [M+H]^+^ ions of 1426.53 and 1400.55 *m/z* respectively in reverse phase LC-MS using electrospray in positive ion mode. The uretdione form of HDI, a stabilized dimer, formed two reaction products with GSH, a tris(GSH)-isocyanate reaction product recognized as a 1258.44 *m/z* [M+H]^+^ ion, and a bis(GSH)-isocyanate product identified as a 951.36 *m/z* [M+H]^+^ ion. Predicted structures for the newly described GSH-polyisocyanate reaction products, modeled based on collision induced dissociation (CID) fragmentation patterns in tandem MS/MS, support *S*-linkage of the GSH to N = C = O groups. In summary, industrially-used aliphatic polyisocyanates readily react with GSH to form primarily *S*-linked tris(GSH)-conjugates, a process that may play an important role in response to respiratory tract exposure.

## Introduction

Isocyanate (N = C = O), a reactive chemical group that readily undergoes nucleophilic addition reactions, is essential to numerous industries [1, 2]. Isocyanate groups attached to aromatic vs. aliphatic carbon backbones have distinct physical properties that influence their reaction products and industrial use [3–6]. Aromatic isocyanates primarily are used to produce solid and flexible polyurethane foam [7, 8], however, its products are susceptible to damage by ultraviolet radiation and oxidation and are prone to discoloration over time [9]. In comparison, aliphatic isocyanates generate products with superior durability under harsh conditions and primarily are used in paint and surface coatings (e.g., automotive, aerospace, military) [10–12]. Aliphatic isocyanates also have specialty applications in solid propellants [13, 14], polymer bonded explosives [13, 15], and medical devices [16, 17].

Adverse health effects from inhaling isocyanate (pulmonary irritation, asthma) are a well-recognized occupational hazard in diverse industries [4, 18]. Workplace isocyanate use requires appropriate personal protective equipment and industrial hygiene to prevent exposure [10, 19]. Endogenous host mechanisms that mediate pathogenic vs. (potentially) protective responses against respiratory tract exposure are unclear but have been suggested to involve glutathione (GSH), a major anti-oxidant of the lower respiratory tract known to play an important role in chemical metabolism [20–25].

The most commonly used aliphatic diisocyanate, hexamethylene diisocyanate (HDI), reacts readily with GSH under physiologic conditions [26]. When HDI is inhaled into the lungs in an animal model, bis(GSH)-HDI is identified as a major product in the airway fluid [27]. In vitro, bis(GSH)-HDI reaction products can be cleaved by human gamma-glutamyl transpeptidase, a critical step along the mercapturic acid pathway of metabolism and elimination [22].

**Fig 1:**
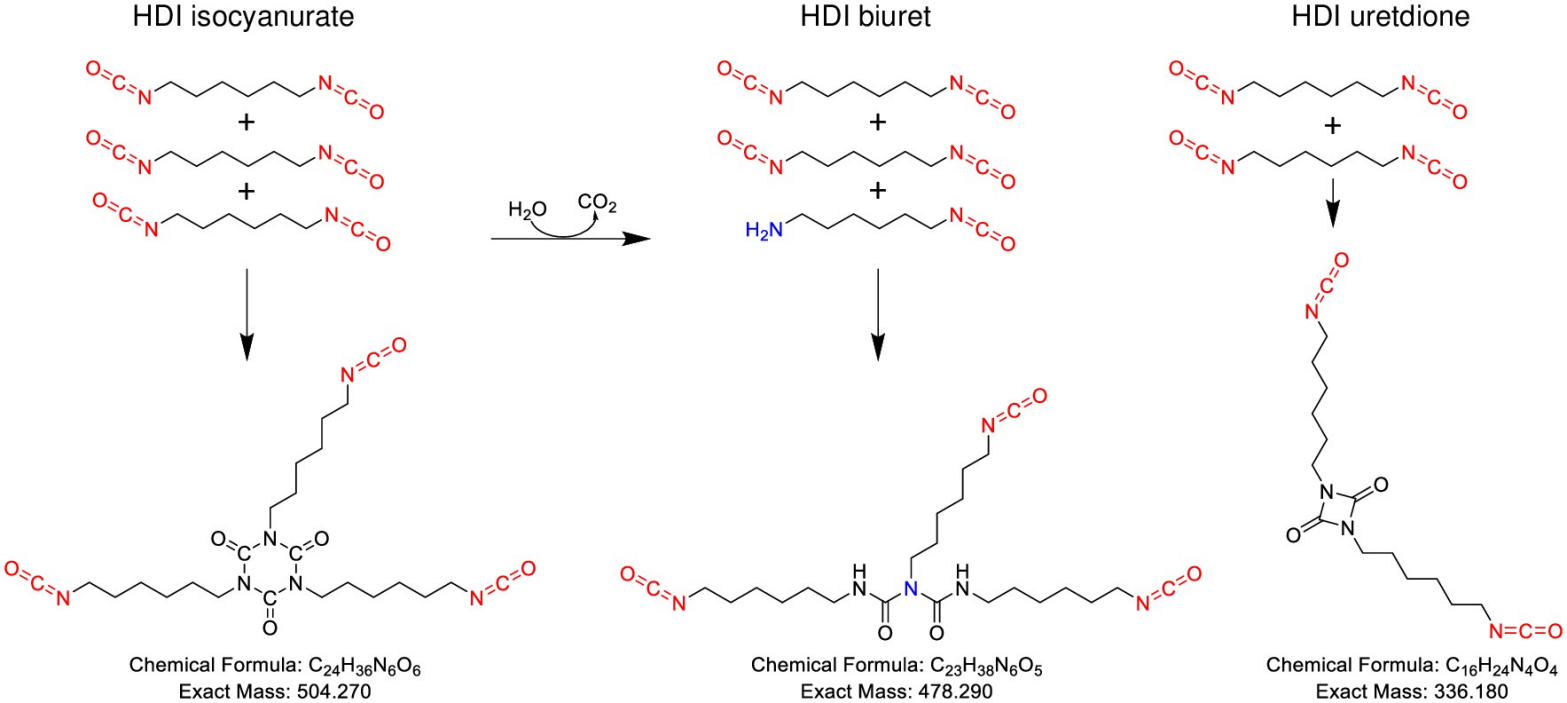
Three most common aliphatic polyisocyanates. Three different polyisocyanates are generated from hexamethylene diisocyanate monomer as shown. The biuret contains one partially hydrolyzed HDI. The 6-member ring structure of the isocyanurate is stable, however one of the internally blocked NCOs of the 4 member ring of the uretdione can react under appropriate conditions.

In contemporary industrial settings HDI has been largely replaced with polymeric hexamethylene diisocyanate formulations, as they have reduced volatility and thus less potential for lower respiratory tract exposure [12]. The most commonly used formulations (Fig 1) are “trimers” of HDI, the isocyanurate and the biuret (prepared with limited addition of water), with slightly different properties that impart distinct advantages/disadvantages in the final product [12, 15]. A “dimeric” form of HDI containing an internally blocked uretdione moiety is also employed for specialty applications and has reduced viscosity making it useful as a “thinning” agent suitable for mixing with other isocyanates [12, 28].

The present investigation explores the reactivity of GSH with aliphatic polyisocyanates most commonly used in industry today. LC-MS and LC-MS/MS are used to characterize reaction products generated under physiologic conditions and model their chemical structures. The findings and their potential implications with regard to protection against isocyanate exposure are discussed.

## Materials and methods

### Aliphatic polyisocyanates

Commercially available preparations of HDI isocyanurate (CAS # 3779-63-3), biuret (CAS # 4035-89-6), and uretdione (CAS # 23501-81-7), the common names for (a) (2,4,6-trioxotriazine-1,3,5(2H,4H,6H)-triyl)tris(hexamethylene) isocyanate, (b) 1,3,5-tris(6-isocyanatohexyl)biuret, and (c) 2,4-dioxo-1,3-diazetidine-1,3-bis(hexamethylene) diisocyanate, were obtained from Covestro (Pittsburgh, PA). HDI biuret was also obtained from Sigma-Aldrich (St. Louis, MO).

### Generation of GSH-isocyanate conjugates

Reduced glutathione, GSH (CAS # 70-18-8), from Sigma-Aldrich was reacted with aliphatic polyisocyanates of HDI as previously described with slight modifications [29]. Briefly, HDI polyisocyanate were diluted 1:10 in acetone to achieve a 10% (v/v) solution, and further diluted 100-fold into GSH solutions to achieve a final reaction concentration of 0.1% (v/v). Ten millimolar GSH solutions were prepared in LC-MS grade water (*note, without buffer GSH solutions are acidic with pH <4) and in solution buffered to pH 7.4 with 200 mM sodium phosphate. The reaction mixture was rotated end-over-end for 2 hours at 37°C, and then centrifuged at 10,000 *g*, and 0.2 μm filtered before analysis.

### LC-MS and LC-MS/MS

Samples were analyzed on a 1290 model Infinity LC system coupled to a 6550 model Q-TOF MS system using a rapid resolution HT Zorbax Eclipse Plus C18 column (2.1 × 50 mm, 1.8 μm); all from Agilent Technologies (Santa Clara, CA) as previously described [30]. For analysis, samples were mixed with water containing 0.1% formic acid, and 3 μL of diluted sample was loaded and eluted with water/0.1% formic acid and increasing concentrations of acetonitrile, also containing 0.1% formic acid. The acetonitrile gradient gradually increased to 40% acetonitrile by 3 minutes, followed by increase to 98% by 4.0 minutes and return to 2% acetonitrile by 5 minutes, although the gradient was extended slightly in studies with HDI uretdione to better separate two reaction products. Positive electrospray ionization (ESI) was performed using the following parameters: gas temp- 280°C, gas flow- 11 l/min, nebulizer-40 psig, sheath gas temp- 350°C, sheath gas flow-11, Vcap-4000 V, nozzle voltage-2000 V, fragmentor voltage– 175 V, skimmer voltage 65 V, octopole RF peak voltage 750 V. For MS/MS analyses, the collision energy was automatically set using Agilent MassHunter Acquisition software according to the formula, slope × (m/z)/100 + offset; with the slope of 5 and offset of 2.5. The m/z values of all ions present in the mass spectra were corrected against two reference ions (purine, [M+H]^+^ m/z 112.9856 and 1H, 1H, 3H tetra(fluoropropoxy)phosphazine, [M+H]^+^ m/z 922.0097). The data acquisition range was from 110–1700 m/z. Quantitation of selected reaction products was based on the area under the curve (AUC) of peaks for [M+H]^+^ ions with defined m/z ratios and retention times in total and extracted ion chromatograms (TICs, EICs) and/or from A210 spectra.

## Data analysis and chemical structures

MassHunter software from Agilent was used for LC-MS and MS/MS acquisition and analysis, including total and extracted ion chromatograms (TIC and EICs), and integration of area under the curve (AUC) for peaks of interest. ChemDraw Professional v.20 was used to model chemical structures and fragmentation patterns and to calculate exact mass of expected products.

## Supporting information studies

Preliminary studies described in S14 and S15 Figs, used pooled IgG from de-identified human serum and animal samples. Human Subject use was approved by the Yale University Human Investigation Committee’s (HIC) Institutional Review Board (IRB), under protocol number 2000027806. Consent was not obtained since the study used de-identified cryopreserved samples. Animal samples were from studies that received ethical approval by Yale University’s Institutional Animal Care and Use Committee (IACUC), protocol number 2021–20076. Animals were sacrificed according to guidelines established by the American Veterinary Medical Association (AVMA), via an intraperitoneal injection of 0.1 mL of Euthasol^®^ (Virbac AH, Inc.; Westlake, TX), a solution containing 390 mg pentobarbital sodium (barbituric acid derivative) and 50 mg phenytoin sodium per mL.

## Results

### HDI isocyanurate

**Fig 2:**
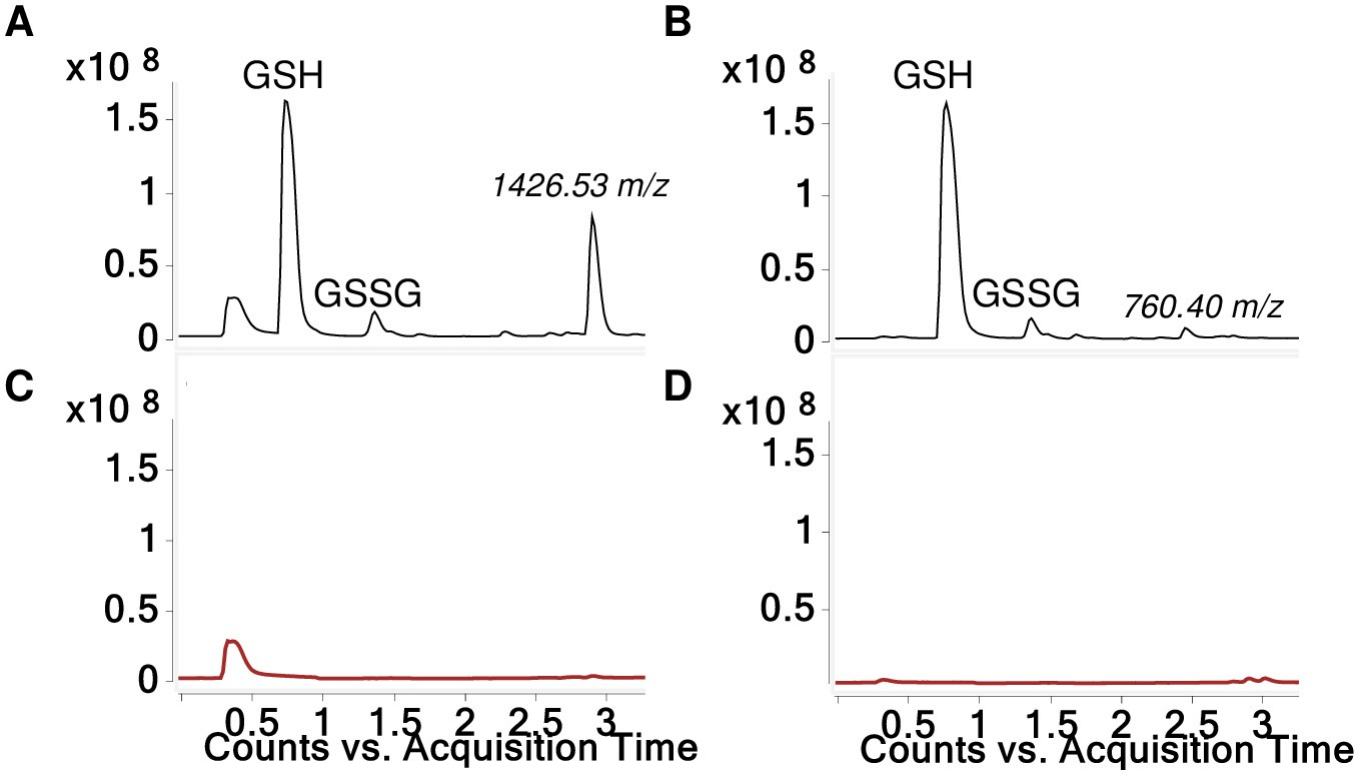
LC-MS analysis of GSH reaction products with HDI isocyanurate. TICs are shown for reaction products generated when HDI isocyanurate was reacted with GSH (A and B) and without GSH (C and D), in solution buffered to pH 7.4 (A and C) or without buffer, pH < 4.0 (B and D). The *m/z* for the major products recognized as new [M+H]^+^ ions are labeled, along with peaks reflecting GSH, GSSG. Unlabeled peak eluting ~0.5 min (A and C) is due to sodium phosphate.

**Fig 3:**
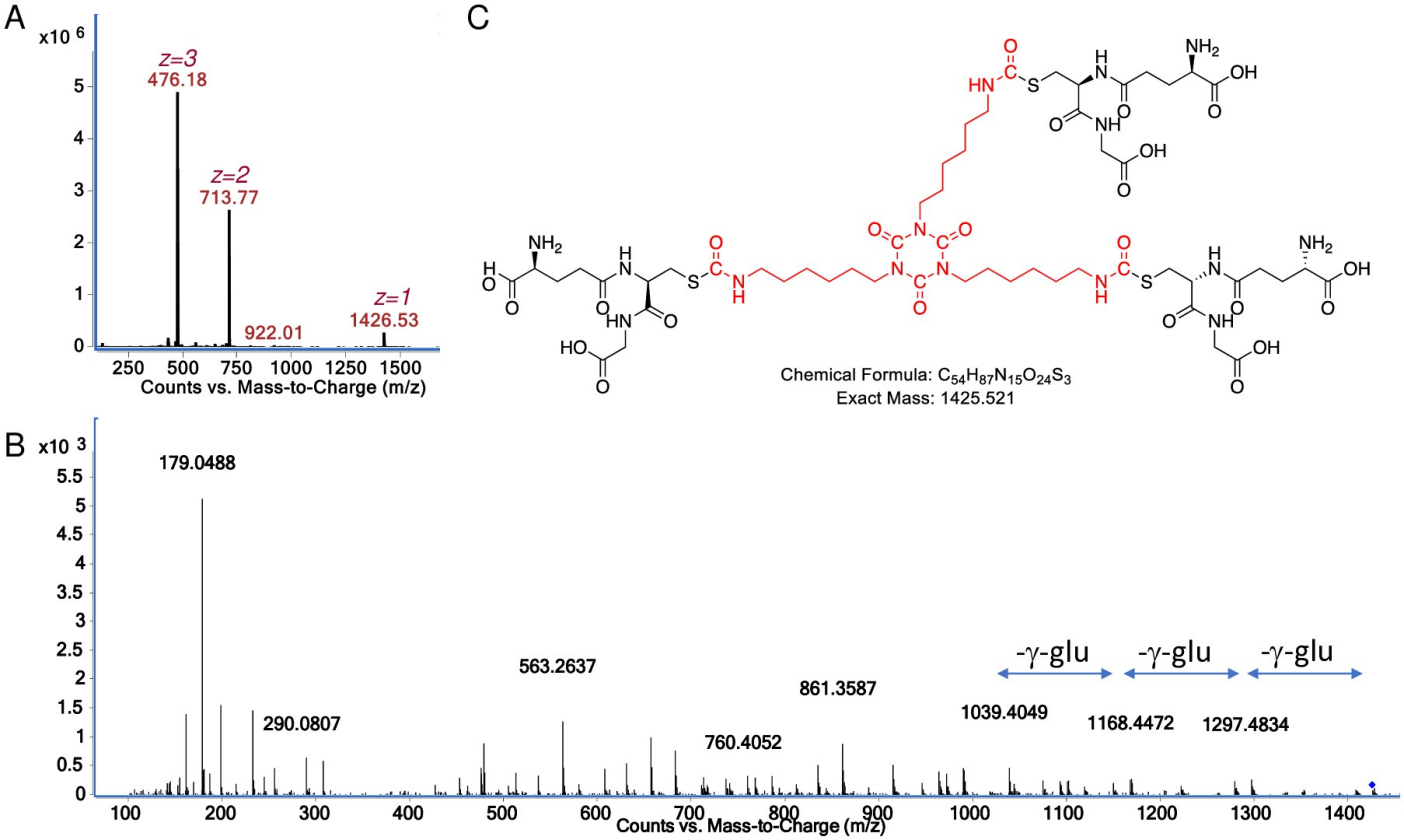
Characterization of GSH reaction product with HDI isocyanurate at physiologic pH. (A) Mass spec analysis of sample eluting from reverse phase LC column ~3 minutes, note dominance of doubly and triply charged species corresponding to the major product, the 1426.53 *m/z* [M+H]^+^ ion. (B) the CID fragmentation spectra of the 1426.53 *m/z* [M+H]^+^ ion upon MS/MS. (C) structural model for the major reaction product of GSH with HDI isocyanurate based on exact mass and expected fragmentation pattern (S2 Fig).

When HDI isocyanurate was reacted with GSH at physiologic pH (7.4) a single reaction product was observed in the LC-MS total ion current (TIC), an [M+H]^+^ ion with a 1426.53 *m/z*, which was detected largely as doubly and triply charged species (Figs 2 and 3). Notably, the 1426.53 *m/z* [M+H]^+^ ion was also the single reaction product detectable quantitatively based on A_210_ absorbance (see S1 Fig). When reactions were carried out without buffer, the pH was < 4.0 due to GSH’s acidity, and limited amounts of a 760.40 *m/z* rather than a 1426.53 *m/z* [M+H]^+^ product was instead observed. In the absence of GSH, very little reaction product was observed, and we speculate HDI isocyanurate likely self-polymerized and became insoluble or exceeded size detection limits for the LC-MS conditions.

Further LC-MS/MS analysis was performed to better characterize the 1426.53 *m/z* [M+H]^+^ product formed when GSH was reacted with HDI isocyanurate at physiologic pH. As shown in Fig 2B, prominent CID fragments are observed corresponding to the neutral loss of 3 gamma-glutamate groups, consistent with a tris, *S*-linked GSH compound. Additional CID fragments expected from CID fragmentation of the tris(GSH)-HDI isocyanurate were prominent in the MS/MS spectra, including the 861 *m/z* [M+H]^+^ GSH-isocyanurate fragment and the 179 *m/z* [M+H]^+^ expected for the cys-gly and other known fragments of GSH (see S2 Fig) [31]. Together the data are consistent with the predicted structure modeled in Fig 2C. LC-MS/MS analysis and chemical structure models for the 760.40 *m/z* [M+H]^+^ that forms when GSH reacts with HDI isocyanurate without buffer (at low pH) are shown in supporting information and are consistent with a single *S*-linked GSH conjugated to a partially hydrolyzed HDI isocyanurate molecule, e.g., two of the three free NCO groups hydrolyzed to primary amines (S3 Fig).

### HDI biuret

**Fig 4:**
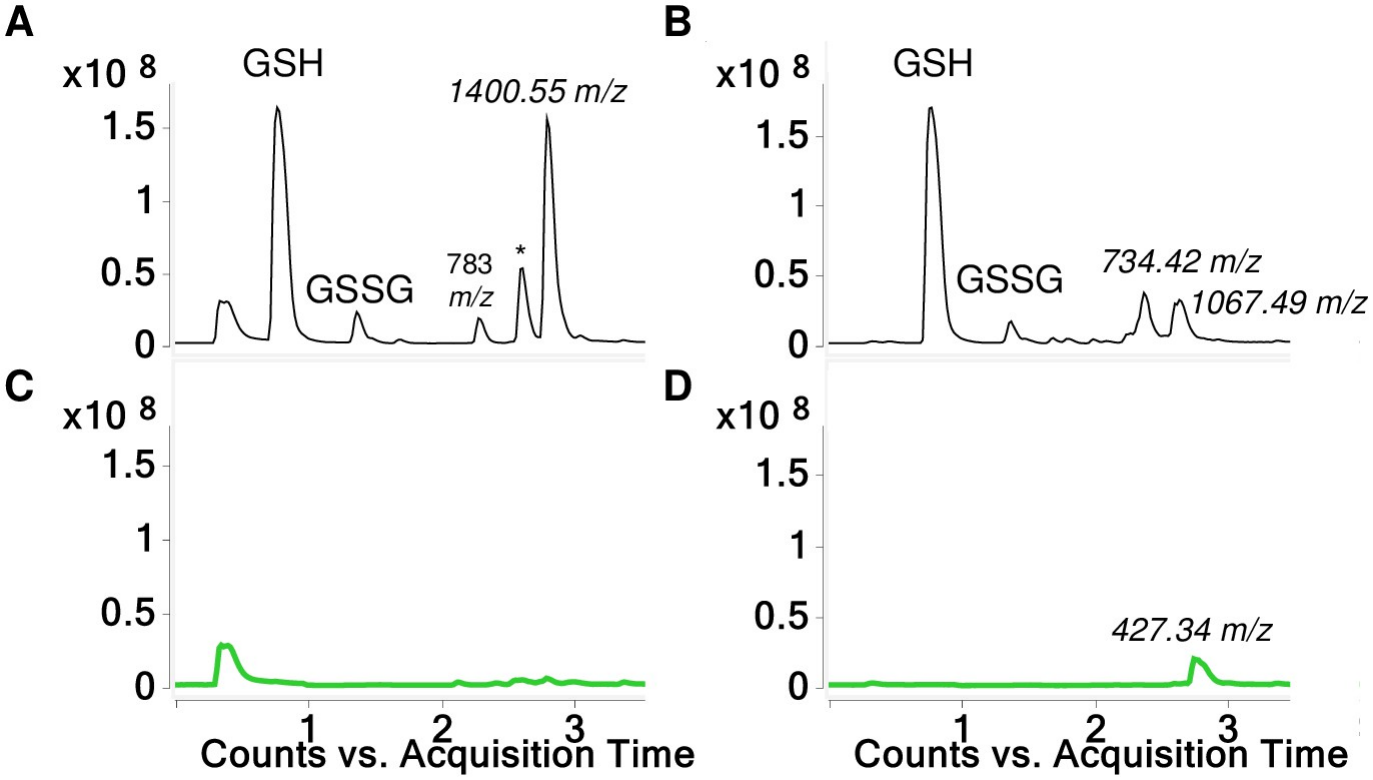
LC-MS analysis of GSH reaction products with HDI biuret. TICs are shown for reaction products generated when HDI biuret was reacted with GSH (A and B) and without GSH (C and D), in solution buffered to pH 7.4 (A and C) or without buffer, pH < 4.0 (B and D). The *m/z* for the major products recognized as new [M+H]^+^ ions are labeled, along with peaks reflecting GSH, GSSG. Unlabeled peak eluting ~0.5 min (A and C) is due to sodium phosphate.

**Fig 5:**
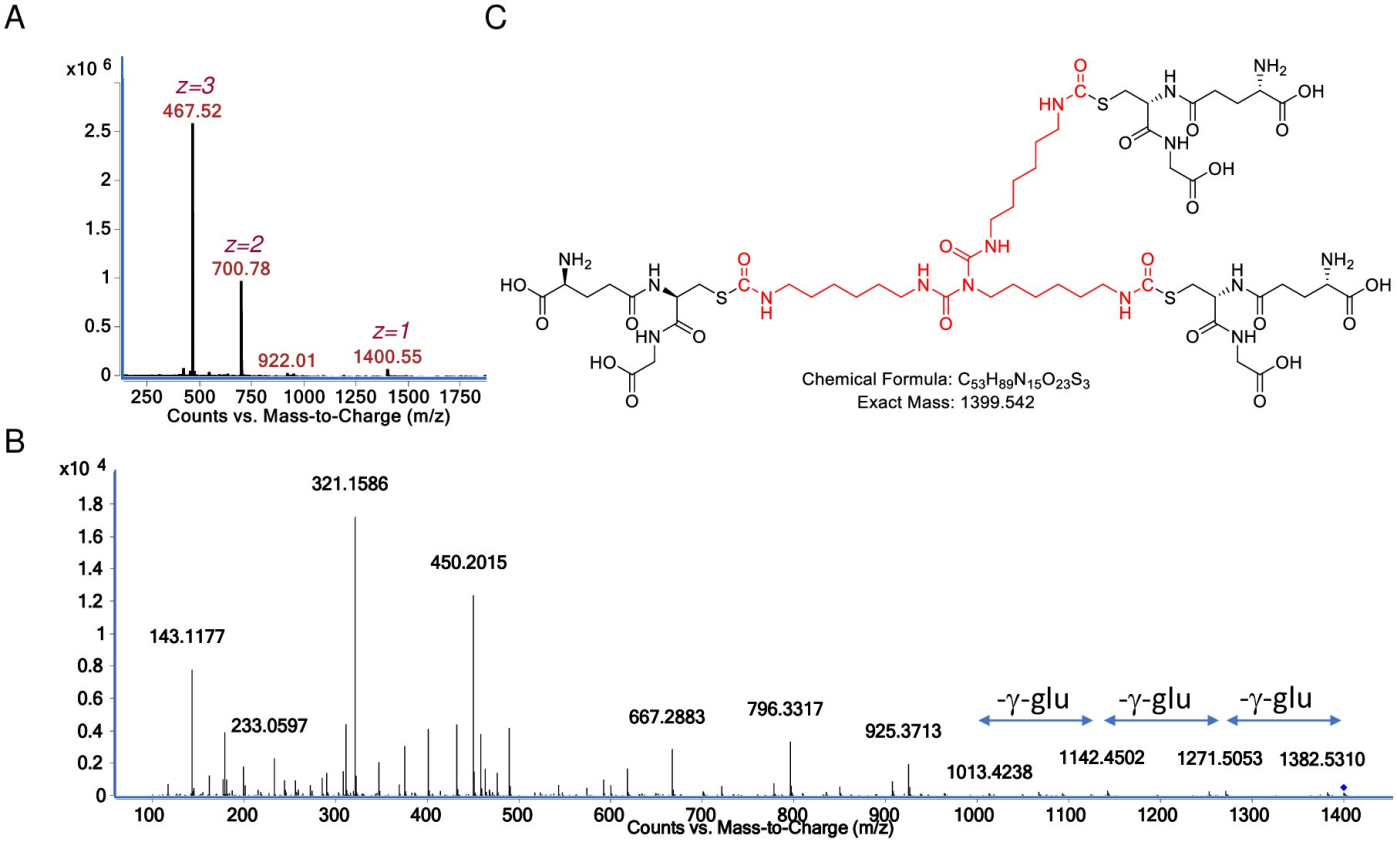
Characterization of major GSH reaction product with HDI biuret at physiologic pH. (A) Mass spec analysis of major product eluting from reverse phase LC column ~2.8 minutes, note dominance of doubly and triply charged species corresponding to the 1400.55 *m/z* [M+H]^+^ ion. (B) the CID fragmentation spectra of the 1400.55 *m/z* [M+H]^+^ ion upon MS/MS. (C) structural model for the major reaction product of GSH with HDI biuret based on exact mass and expected fragmentation pattern (S6 Fig).

When commercial HDI biuret was reacted with GSH at physiologic pH (7.4) three reaction products were observed in the LC-MS total ion current (TIC). The dominant product was an [M+H]^+^ ion with a 1400.55 *m/z*, which was detected primarily as doubly and triply charged species (Figs 4 and 5). A minor product eluting at 2.3 minutes was a 783.26 *m/z* [M+H]^+^ (S4 Fig) consistent with that previously described for bis(GSH)-HDI [26] and the known presence of low level HDI monomer in commercial biuret products. A third product, a 1258.44 *m/z* [M+H]^+^ ion, was initially unexpected and is described in more detail below (see HDI uretdione section). Notably, the major reaction products of HDI biuret with GSH under physiologic conditions observed in the TIC were proportionally observed in the A_210_ spectra (S5 Fig), with ratios of 1:3:12, for the 783.26, 1258.44 and 1400.55 *m/z* [M+H]^+^ species respectively, deduced by integration of the AUC for the corresponding peaks.

When reactions were carried out without buffer, limited amounts of 734.42 and 1067.49 *m/z* [M+H]^+^ products were observed in contrast to the major 1400.55 *m/z* [M+H]^+^ ion formed at physiologic pH. In the absence of GSH at pH 7.4 very little reaction product was observed, however without buffer a 427.34 *m/z* [M+H]^+^ was detected, consistent with partial hydrolysis followed by intramolecular-reactivity of the biuret (see S7 Fig).

Further LC-MS/MS analysis was performed to better characterize the major 1400.55 *m/z* [M+H]^+^ product formed when GSH was reacted with HDI biuret at physiologic pH. As shown in Fig 5, prominent CID fragments are observed corresponding to the neutral loss of 3 gamma-glutamate groups from intact tris(GSH)-HDI biuret. Additional CID fragments expected from an *S*-linked tris(GSH)-HDI biuret were prominent in the MS/MS spectra, including 321.15 and 450.20 *m/z* [M+H]^+^ ions expected from GSH-HDI biuret fragments, the 143.12 m/z expected from one partial HDI subunit, and 179.05 and 233.06 *m/z* [M+H]^+^ ions expected for GSH (cys-gly and glu-cys) fragments (S6 Fig). Together the data are consistent with the predicted tris(GSH)-HDI biuret structure modeled in Fig 5c.

LC-MS/MS analysis of the 734.42 and 1067.49 *m/z* [M+H]^+^ products that formed when GSH was reacted with HDI biuret in solution without buffer (pH <4) was consistent with those expected for mono and bis(GSH) conjugated to partially hydrolyzed HDI biuret, e.g., with hydrolysis of the non-GSH bound NCO(s) to primary amine(s). MS/MS data and proposed structures are provided in S8 and S9 Figs.

### HDI uretdione

**Fig 6:**
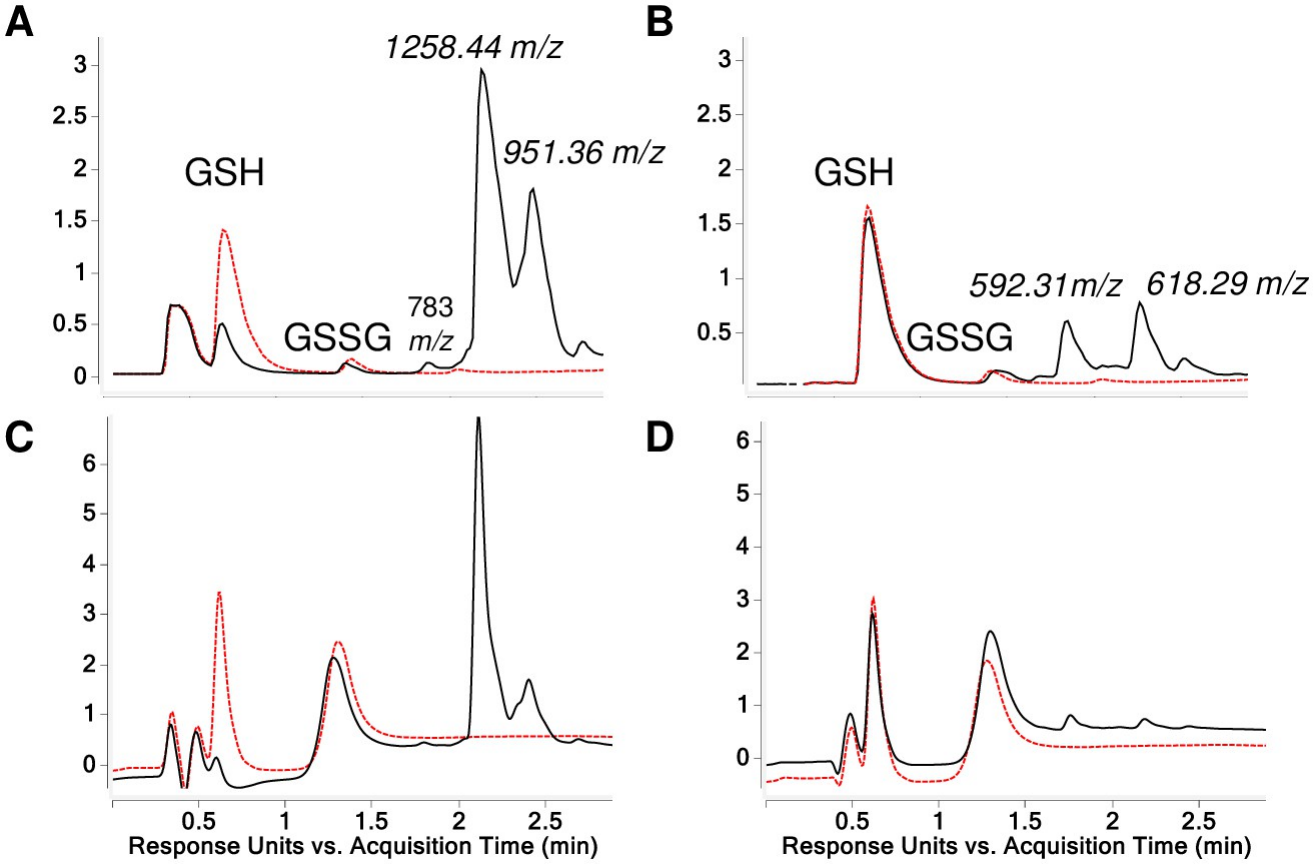
LC-MS analysis of GSH reaction products with HDI uretdione. TICs (A and B) and A_210_ chromatograms (C and D) are shown for reaction products generated when GSH was reacted with HDI uretdione (solid black line) or without (dashed red line). The *m/z* for the major products recognized as new [M+H]^+^ ions are labeled, along with GSH and GSSG.

**Fig 7:**
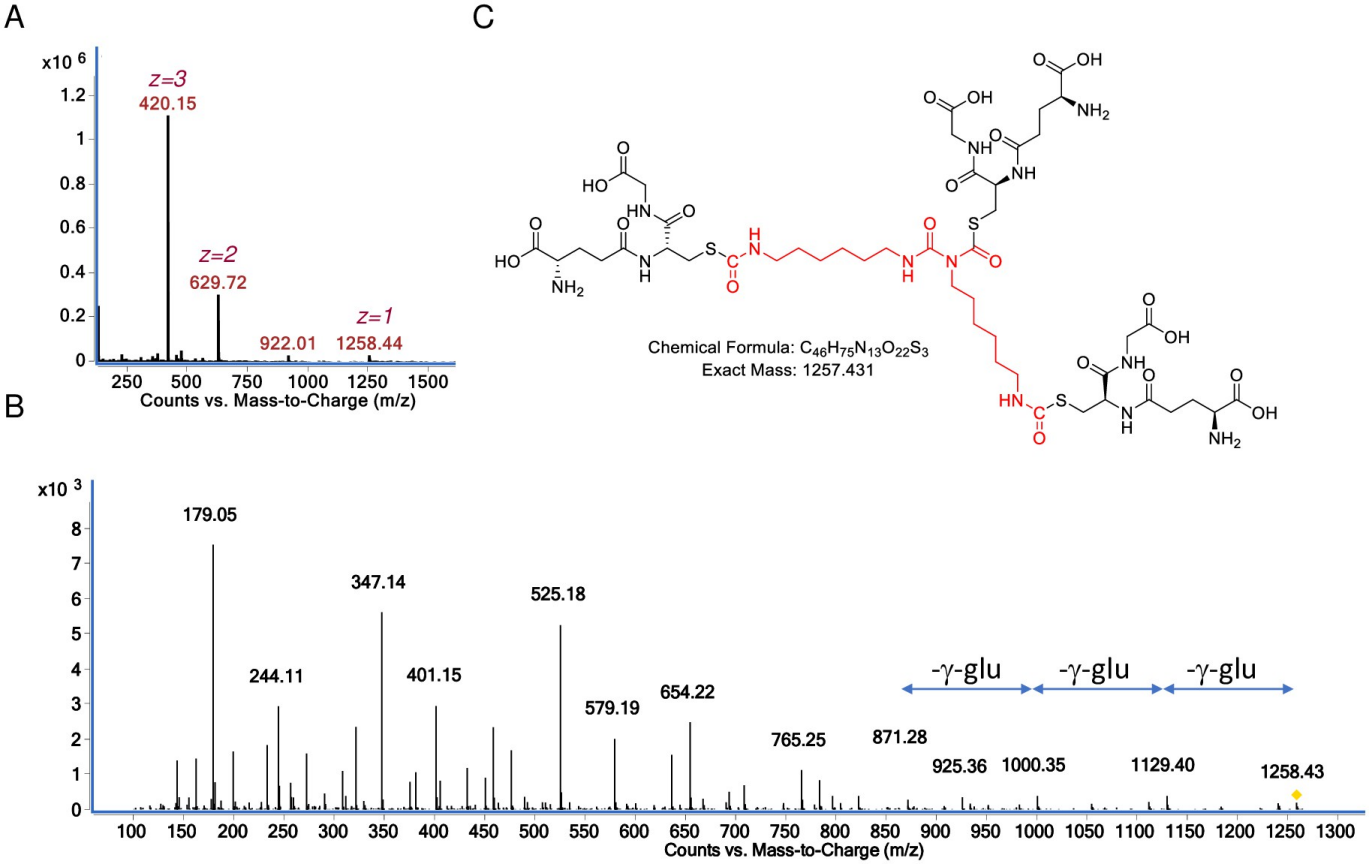
Characterization of major GSH reaction product with HDI uretdione at physiologic pH. (A) Mass spec analysis of sample eluting from reverse phase LC column ~ 2.1 minutes, note dominance of doubly and triply charged species corresponding to the 1258.44 *m/z* [M+H]^+^ ion. (B) the CID fragmentation spectra of the 1258.44 *m/z* [M+H]^+^ ion upon MS/MS. (C) structural model for the major reaction product of GSH with HDI uretdione based on exact mass and expected fragmentation pattern (S10 Fig).

**Fig 8:**
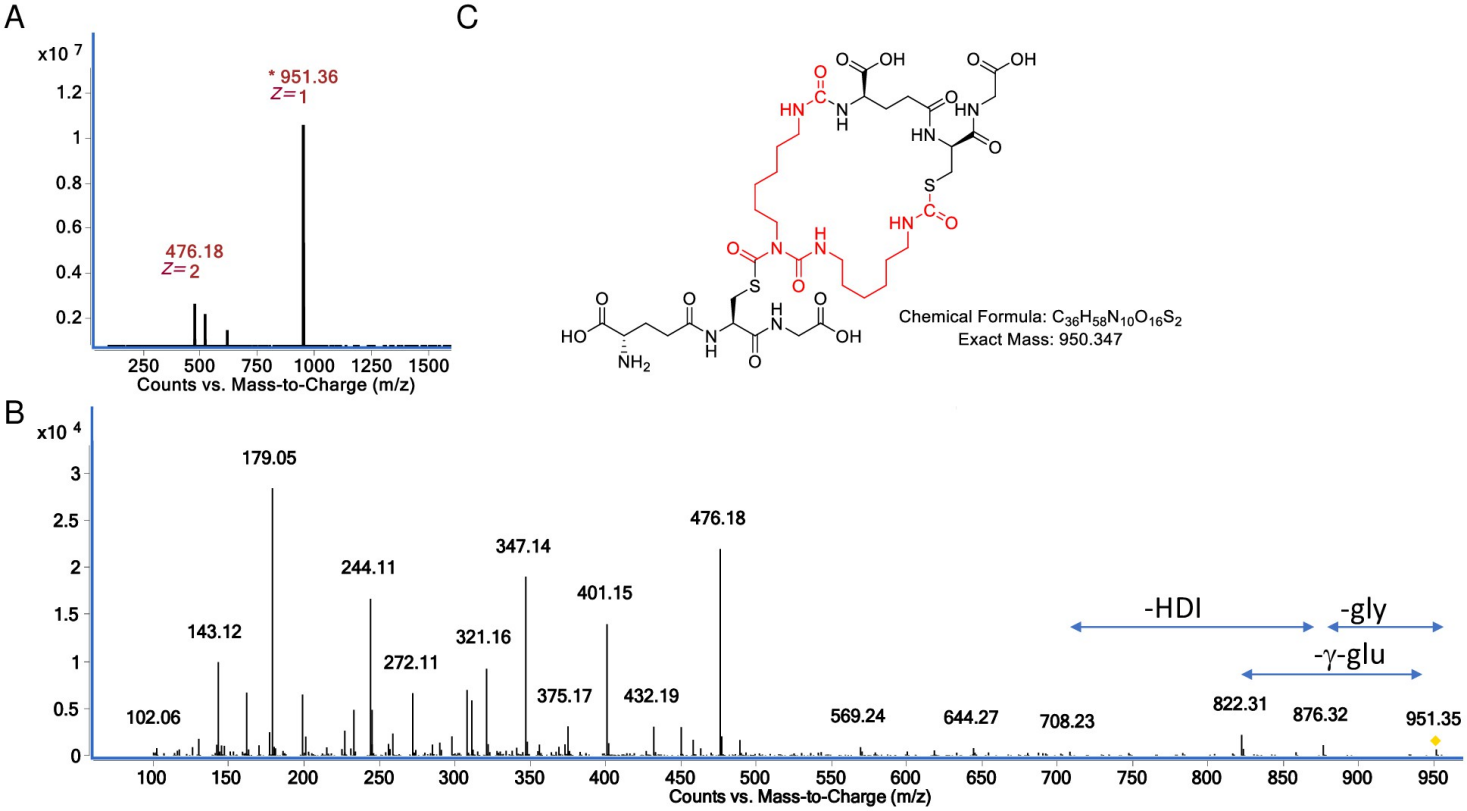
Characterization of minor GSH reaction product with HDI uretdione at physiologic pH. (A) Mass spec analysis of sample eluting from reverse phase LC column ~ 2.3 minutes, note dominance of doubly and triply charged species corresponding to the 951.36 *m/z* [M+H]^+^ ion. (B) the CID fragmentation spectra of the 951.36 *m/z* [M+H]^+^ ion upon MS/MS. (C) structural model for the minor reaction product of GSH with HDI uretdione based on exact mass and expected fragmentation pattern (S11 Fig).

HDI uretdione is formed from two HDI ‘monomers” and contains 2 free NCO groups and 2 additional NCO groups that exist in equilibrium as a cyclized moiety, essentially creating an internally blocked NCO. While technically a HDI dimer, the uretdione exhibits trifunctionallity as one of the internally stabilized NCO groups can undergo nucleophilic addition under appropriate conditions. When commercial HDI uretdione was reacted with GSH at physiologic pH 7.4, two major reaction products were observed in the TIC (Figs 6A, 7 and 8). The dominant products, 1258.44 and 951.36 *m/z* [M+H]^+^ ions, were present at a 9:1 ratio based on corresponding peaks in the A_210_ spectra. In contrast, when HDI uretdione was reacted with GSH without buffer (pH <4) quantitatively limited reaction products observed in A_210_ spectra corresponded to 592.31 and 618.29 *m/z* [M+H]^+^ products (Fig 6B).

Further LC-MS/MS analysis was performed to better characterize the major 1258.44 and 951.36 *m/z* [M+H]^+^ products that formed when GSH was reacted with HDI uretdione at physiologic pH. As shown in Fig 7B for the1258.44 *m/z* [M+H]^+^ ion, CID fragments are observed corresponding to the neutral loss of 3 gamma-glutamate groups, consistent with an *S*-linked (tris)GSH compound. Additional CID fragments expected from CID fragmentation of tris(GSH)-HDI uretdione were prominent in the MS/MS spectra, including the 925.36, 765.25, 654.22. 525.18, 401.15, and 347.14 *m/z* [M+H]^+^ ions, along with the 179 *m/z* [M+H]^+^ expected for the cys-gly portion of GSH (see S10 Fig). Together the data support the structure for tris(GSH)-HDI uretdione presented in Fig 7C, however alternative conformations cannot be definitively ruled out from the data.

LC-MS/MS data (Fig 8B) on the 951.36 *m/z* [M+H]^+^ ion was analyzed to better characterize this minor product. Notably, the CID spectra contained a 708.23 *m/z* [M+H]^+^ ion, consistent with neutral loss of one HDI (168.09 amu) and one gly moiety (75.03 amu), as described in (S11 Fig). Such a fragment would likely only arise from a compound where one GSH conjugation site is on the uretdione ring and the 2 terminal N = C = O groups are stabilized (without changing the mass) by cross-linking a second GSH group, as shown in Fig 8C. The CID spectra of the 951.36 *m/z* [M+H]^+^ ion contained numerous other fragments consistent with a bis(GSH)-uretdione, including 644.27, 569.24, 476.18, 401.18, 347.14, 321.16, 179.05, and 143.12 *m/z* [M+H]^+^ ions.

LC-MS/MS analysis was also performed on the of 592.31 and 618.29 *m/z* [M+H]^+^ products that formed when GSH was reacted with HDI uretdione in solution without buffer (pH <4). The data (S12 and S13 Figs) are consistent with mono(GSH) conjugates with partially hydrolyzed HDI uretdione, either with two NCO groups hydrolyzed to amines or with one NCO group hydrolyzed and one NCO group bound to GSH’s amino terminus, possibly in a cyclized manner as previously described for di-isocyanates [21, 26].

## Discussion

GSH, an important mediator of xenobiotic metabolism, was shown to react with isocyanates of the aliphatic polymeric type used most commonly in making coatings and elastomers. LC-MS/MS data demonstrate reactivity of GSH with free NCOs in trimers of HDI (isocyanurate and biuret) resulting in *S*-linked tris(GSH) reaction products at physiologic pH. Tris(GSH) reaction products, and to a lesser extent bis(GSH) were also observed with dimerized HDI formulations that contain two free NCO groups and an additional two NCO groups stabilized in a uretdione moiety (e.g., internally blocked), consistent with multi-functionality of the chemical recognized industrially. The data highlight the reactivity of isocyanate groups on distinct organic backbones, including those stabilized in uretdione structures, with the unique thiol in the cysteine of GSH.

Reactivity of GSH with aliphatic polyisocyanates extends observations of studies with di-isocyanates (both aromatic and aliphatic) and suggests a common susceptibility of NCO compounds toward reactivity with GSH’s uniquely reactive thiol. GSH concentrations are relatively high in the lower airway [25], the primary site for isocyanate toxicity [32], and most commonly, but not always, protect self-molecules (DNA, proteins) against alkylating electrophilic xenobiotics [24, 33]. Further understanding of the fate of GSH-isocyanate adducts in exposed airways in vivo, as recently described for monomeric MDI [21, 34, 35], may help understand chemical metabolism and potentially pathogenic outcomes of exposure.

The reaction pH was found to markedly influence the reaction between polyisocyantes and GSH. In the absence of buffer the reaction pH was low (pH < 4) due to GSH’s innate acidity and tris(GSH) reaction products were not observed. Instead, we observed evidence for isocyanate hydrolysis and/or [M+H]^+^ ions expected for intramolecularly-reacted (cyclized) products, with *S-* and *N*-linkage of a single GSH molecules, as previously reported with HDI “monomer” [21, 26]. In the absence of GSH or other reactive compounds, reactivity of NCO groups with water (hydrolysis) likely resulted in free amine groups, with subsequent polymerization to products above detection limits of the LC-MS methodology or which precipitated and were filtered from solution before final analysis. HDI biuret was the exception among the three polyisocyanates tested; in water it yielded small amounts of a low molecular weight (427 *m/z* [M+H]^+^) ion with characteristics of an intramolecularly-linked (cyclized) urea.

Characteristics of the reaction products and methodology are important aspects of the present study. The workup conditions of the LC-MS (dilution in formic acid buffer) likely stabilized the thiol-isocyanate reaction products, which can undergo hydrolysis at higher pH. The analytical conditions also highlighted trimeric reaction products in LC-MS, which were detected primarily as triply charged species, consistent with trivalent adducts. CID fragmentation patterns in LC-MS/MS analysis provided further evidence for *S*- linkage of GSH, most notably, loss of g-glutamyl groups as described for other GSH conjugates [31].

The strengths and weakness of the study should be recognized in considering the significance of the findings. A major strength was the use of highly precise LC-MS/MS techniques to identify and characterize GSH reaction products with aliphatic polyisocyanates, which support chemical structural models. The major weakness of the study is the ex-vivo nature of the experiments. While the reaction products were generated under physiologic pH, their stability and potential ability to subsequently transfer polyisocyanate to self-molecules remains unclear. Preliminary data from ongoing studies in our laboratory suggest GSH-polyisocyanate can transfer polyisocyanate to (carbamylate) albumin in vitro and in vivo, creating antigenic changes recognized by serum IgG specifically from exposed workers and anti-aliphatic isocyanate mAbs (S14 and S15 Figs). However, the precise role of GSH in protective (vs pathogenic) responses to occupational isocyanate exposure remains unclear to date.

A minor weakness of the study is its qualitative design, which was not intended to compute mass-balance or quantitate differences in GSH reactivity with different polyisocyanates. The three isocyanate oligomers under study are liquids and were used at constant volumes (as commonly done industrially, specifically 0.1% which is slightly above the regulatory limit established by the European Union), but which differed slightly in molarity [4, 36]. None-the-less, substantially greater reactivity of GSH with (uretdione) HDI dimer vs. trimers (beyond that expected from molar difference) was suggested based on A_210_ absorbance spectra of reaction products, which showed greater decrease in reactant (GSH) and increase in total GSH-isocyanate reaction products.

In summary, the present data extend our understanding of isocyanate reactivity with GSH, a molecule of vital importance in human health and major anti-oxidant in the fluid that lines the lower airways and helps protect against inhaled toxicants. The findings demonstrate the ready reactivity under physiologic conditions of GSH’s unique thiol with multiple NCO groups attached to diverse aliphatic backbones of varying size/structure, including the “internally blocked” NCO groups of HDI uretdione. The data are consistent with studies demonstrating GSH as a primary reactant in vivo for aliphatic HDI (which serves as the building block for the aliphatic polyisocyanates studied herein), methyl isocyanate (cause of the “Bhopal” chemical disaster), and 2-chloroethyl-isocycanate (a metabolic bi-product of an experimental cancer drug). Further studies will help define the potentially central role of GSH in response to NCO exposure, whether from occupational (di-, poly-isocyanate), or recently recognized environmental sources (e.g., hydrogen isocyanate) [37, 38].

## Supporting information

S1 Fig Comparison of TIC and A_210_ spectra for GSH reaction products with HDI isocyanurate at pH 7.4. The TIC (red dashed line) and A_210_ spectra (black solid line) of end-products from GSH reaction with HDI isocyanurate are overlayed and normalized to the highest peak in each spectrum (GSH). The major new peak in the A_210_ spectra when GSH is reacted with HDI isocyanurate corresponds to the 1426.53 *m/z* [M+H]^+^ in the TIC.
(PDF)

S2 Fig Expected fragmentation pattern for tri(GSH)-HDI isocyanurate upon CID in MS/MS. The [M+H]^+^ ions predicted to result from CID of the major reaction product of GSH with HDI isocyanurate under physiologic pH.
(PDF)

S3 Fig Characterization of major GSH reaction product with HDI isocyanurate in solution without pH buffer (i.e., pH < 4). (A) Mass spec analysis of sample eluting from reverse phase LC column ~ 2.6 minutes (i.e., the major reaction product without buffer) and (B) structural model for this major reaction product of GSH with HDI isocyanurate that occurs in the absence of pH buffer (i.e., pH < 4.0) based on exact mass and fragmentation pattern.
(PDF)

S4 Fig LC-MS of minor GSH reaction product with HDI biuret at physiologic pH. The minor product of GSH with commercial HDI biuret exhibits characteristics consistent with GSH-HDI (monomer).
(PDF)

S5 Fig Comparison of TIC and A_210_ spectra for GSH reaction products with HDI biuret at pH 7.4. The TIC (red dashed line) and A_210_ spectra (black solid line) of end-products from GSH reaction with HDI biuret are overlayed and normalized to the highest peak in each spectrum (GSH). The major new peak in the A210 spectra when GSH is reacted with HDI biuret corresponds to the 1400.55 *m/z* [M+H]^+^ in the TIC.
(PDF)

S6 Fig Expected fragmentation pattern for tri(GSH)-HDI biuret upon CID in MS/MS. The [M+H]^+^ ions predicted to result from CID of the major reaction product of GSH with HDI biuret under physiologic pH.
(PDF)

S7 Fig LC-MS of dominant peak when HDI biuret is “reacted” in water (without buffer). The minor product of HDI biuret following control reaction in water without GSH or buffer possess characteristics consistent with the intramolecularly reacted product shown.
(PDF)

S8 Fig Characterization of GSH reaction products with HDI biuret in solution without pH buffer (i.e., pH < 4). (A) Mass spec analysis of sample eluting from reverse phase LC column ~2.4 minutes. (B) the CID fragmentation spectra of the 734.42 *m/z* [M+H]^+^ ion upon MS/MS. (C) structural model for one reaction product of GSH with HDI biuret that occurs in the absence of pH buffer (i.e., pH < 4.0) based on exact mass and fragmentation pattern.
(PDF)

S9 Fig Characterization of GSH reaction products with HDI biuret in solution without pH buffer (i.e., pH < 4). (A) Mass spec analysis of sample eluting from reverse phase LC column ~2.6 minutes. (B) the CID fragmentation spectra of the 1067.49 *m/z* [M+H]^+^ ion upon MS/MS. (C) structural model for a second reaction product of GSH with HDI biuret that occurs in the absence of pH buffer (i.e., pH < 4.0) based on exact mass and expected fragmentation pattern.
(PDF)

S10 Fig Expected fragmentation pattern for tris(GSH)-HDI uretdione upon CID in MS/MS. The [M+H]^+^ ions predicted to result from CID of the major reaction product of GSH with HDI uretdione under physiologic pH.
(PDF)

S11 Fig Expected fragmentation pattern for bis(GSH)-HDI uretdione upon CID in MS/MS. The [M+H]^+^ ions predicted to result from CID of the minor reaction product of GSH with HDI uretdione under physiologic pH.
(PDF)

S12 Fig Characterization of GSH reaction products with HDI uretdione in solution without pH buffer (i.e., pH < 4). (A) Mass spec analysis of sample eluting from reverse phase LC column ~ 1.72 minutes. (B) the CID fragmentation spectra of the 592.31 *m/z* [M+H]^+^ ion upon MS/MS. (C) structural model for one reaction product of GSH with HDI uretdione that occurs in the absence of pH buffer (i.e., pH < 4.0) based on exact mass and fragmentation pattern.
(PDF)

S13 Fig Characterization of GSH reaction products with HDI uretdione in solution without pH buffer (i.e., pH < 4). (A) Mass spec analysis of sample eluting from reverse phase LC column ~ 2.2 minutes. (B) the CID fragmentation spectra of the 618.29 *m/z* [M+H]^+^ ion upon MS/MS. (C) structural model for a second reaction product of GSH with HDI uretdione that occurs in the absence of pH buffer (i.e., pH < 4.0) based on exact mass and expected fragmentation pattern.
(PDF)

S14 Fig Carbamylating capacity of GSH-HDI isocyanurate reaction products in vitro. Human albumin was co-incubated with control (lanes 1, 3, 5) or GSH-HDI isocyanurate reaction products (lanes 2, 4, 6) overnight at 37°C, pH 9.0 followed by SDS-PAGE under reducing conditions and Coomassie blue staining or Western blotting with pooled serum IgG from unexposed individuals or HDI isocyanurate exposed workers as labeled. Note shift in electrophoretic migration of albumin following co-incubation with GSH-HDI isocyanurate consistent with conformational change that is recognized specifically by pooled serum IgG from exposed workers. (B) Right side shows hypothetical carbamylation of human albumin by GSH-isocyanurate reaction product, resulting in stable conjugation to lysine residues. Di-lysine motifs of albumin are preferred reaction sites for di-isocyanates, including “monomeric” HDI vapor [21, 27].
(PDF)

S15 Fig Carbamylating capacity of GSH-HDI uretdione reaction products in vivo. Bronchoalveolar lavage fluid from different mice given GSH-HDI uretdione reaction products (EU) or controls (C), once daily X 5 days, were western blotted with a mAb that specifically recognizes aliphatic isocyanate conjugated proteins (Panel A). The band at ~68 kDa likely reflects albumin, the best recognized “carrier” protein for diisocyanates in vivo and dominant airway fluid protein. Western blots were negative with polyclonal antibody that specifically recognizes HDI (monomer)-conjugated proteins (not shown) and control murine IgM (Panel B). *Note BAL fluid was depleted of immunoglobulin using protein G, mice were B-cell deficient, and anti-aliphatic isocyanate mAb is IgM isotype.
(PDF)
