# Supplementary material for: Glutathione reactivity with aliphatic polyisocyanates
Source: PLoS One. 2022 Jul 15;17(7):e0271471. doi: 10.1371/journal.pone.0271471 (PMC9286259; doi:10.1371/journal.pone.0271471)
Supplement: S1 Fig — The TIC (red dashed line) and A210 spectra (black solid line) of end-products from GSH reaction with HDI isocyanurate are overlayed and normalized to the highest peak in each spectrum (GSH). The major new peak in the A210 spectra when GSH is reacted with HDI isocyanurate corresponds to the 1426.53 m/z [M+H]+ in the TIC. (PDF) [file pone.0271471.s001.pdf]

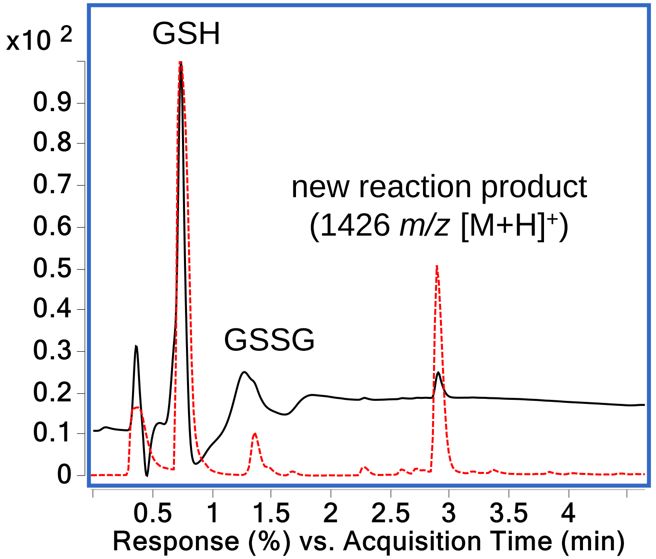

S1 Fig. Comparison of TIC and A210 spectra for GSH reaction products with HDI isocyanurate at pH 7.4. The TIC (red-dashed line) and A210 spectra (black-solid line) of end-products from GSH reaction with HDI isocyanurate are overlayed and normalized to the highest peak in each spectra (GSH). The major new peak resulting from GSH reactivity in the A210 spectra corresponds to the 1426.53 *m/z* [M+H]+ in the TIC.
