# Supplementary material for: Glutathione reactivity with aliphatic polyisocyanates
Source: PLoS One. 2022 Jul 15;17(7):e0271471. doi: 10.1371/journal.pone.0271471 (PMC9286259; doi:10.1371/journal.pone.0271471)
Supplement: S3 Fig — (A) Mass spec analysis of sample eluting from reverse phase LC column ~ 2.6 minutes (i.e., the major reaction product without buffer) and (B) structural model for this major reaction product of GSH with HDI isocyanurate that occurs in the absence of pH buffer (i.e., pH < 4.0) based on exact mass and fragmentation pattern. (PDF) [file pone.0271471.s003.pdf]

A

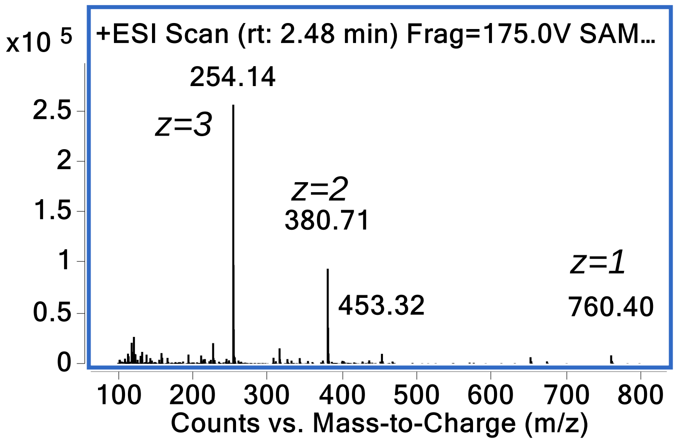

B

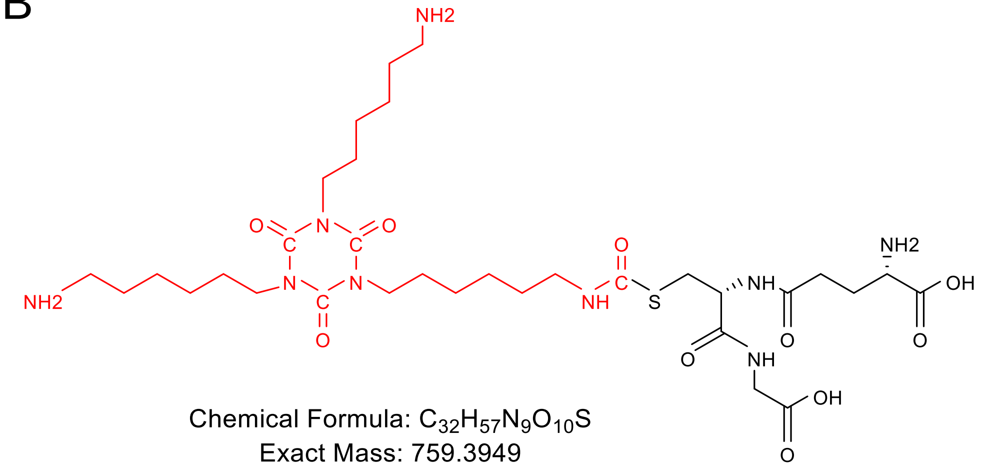

S3 Fig. Characterization of major GSH reaction product with HDI isocyanurate in solution without pH buffer (i.e. pH < 4). (A) Mass spec analysis of sample eluting from reverse phase LC column ~ 2.6 minutes (i.e. the major reaction product without buffer) and (B) structural model for this major reaction product of GSH with HDI isocyanurate that occurs in the absence of pH buffer (i.e. pH < 4.0) based on exact mass and fragmentation pattern (not shown).
