# Supplementary material for: Glutathione reactivity with aliphatic polyisocyanates
Source: PLoS One. 2022 Jul 15;17(7):e0271471. doi: 10.1371/journal.pone.0271471 (PMC9286259; doi:10.1371/journal.pone.0271471)
Supplement: S4 Fig — The minor product of GSH with commercial HDI biuret exhibits characteristics consistent with GSH-HDI (monomer). (PDF) [file pone.0271471.s004.pdf]

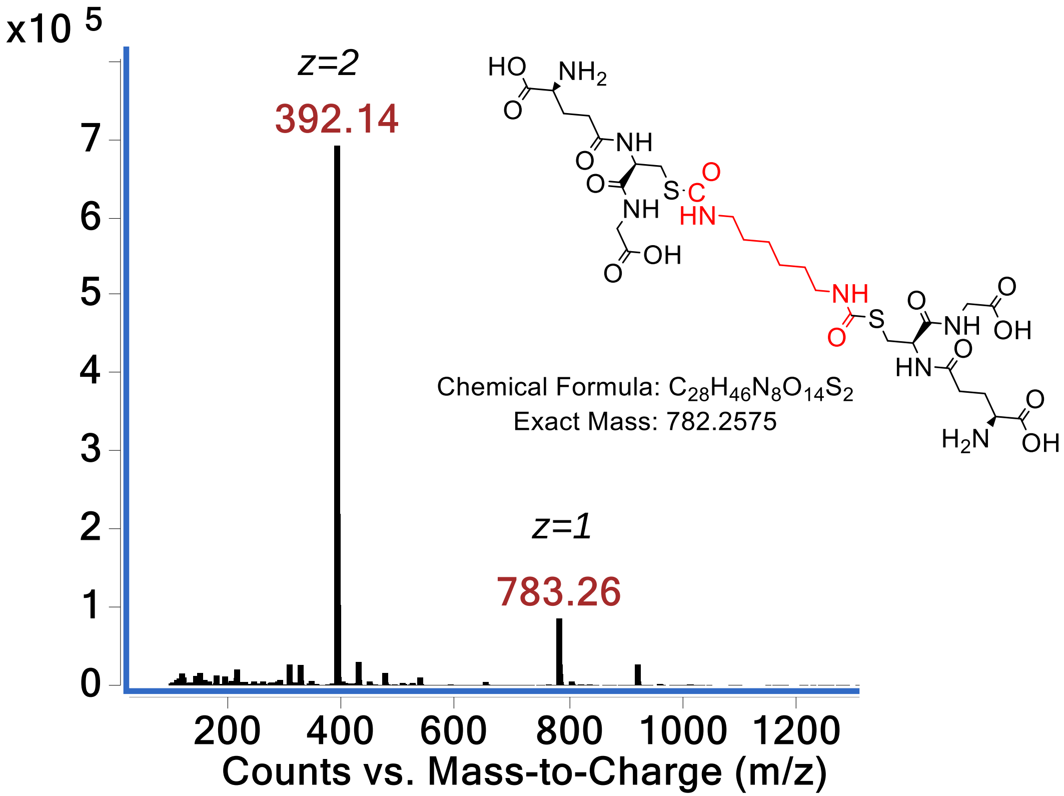

S4 Fig. LC-MS of minor GSH reaction product with HDI biuret at physiologic pH. The minor product of GSH with commercial HDI biuret exhibits characteristics consistent with GSH-HDI (monomer) as we have previously published.
