# Supplementary material for: Glutathione reactivity with aliphatic polyisocyanates
Source: PLoS One. 2022 Jul 15;17(7):e0271471. doi: 10.1371/journal.pone.0271471 (PMC9286259; doi:10.1371/journal.pone.0271471)
Supplement: S5 Fig — The TIC (red dashed line) and A210 spectra (black solid line) of end-products from GSH reaction with HDI biuret are overlayed and normalized to the highest peak in each spectrum (GSH). The major new peak in the A210 spectra when GSH is reacted with HDI biuret corresponds to the 1400.55 m/z [M+H]+ in the TIC. (PDF) [file pone.0271471.s005.pdf]

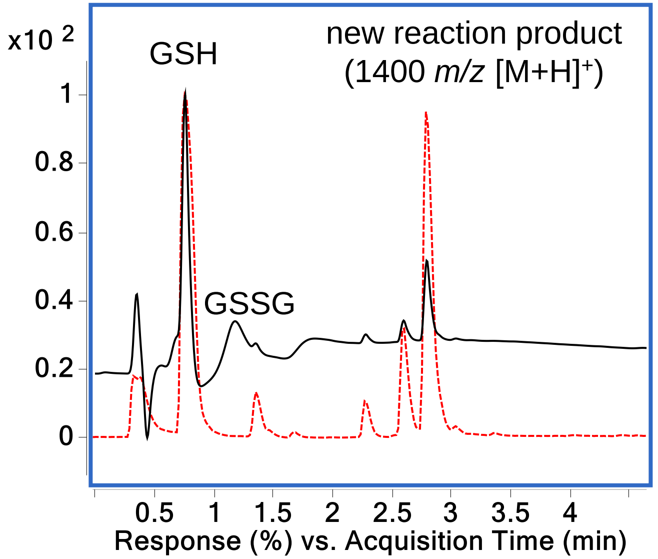

S5 Fig. Comparison of TIC and A210 spectra for GSH reaction products with HDI biuret at pH 7.4. The TIC (red dashed line) and A210 spectra (black solid line) of end-products from GSH reaction with HDI biuret are overlayed and normalized to the highest peak in each spectra (GSH). The quantitatively major new peak in the A210 spectra corresponds to the 1400.55 *m/z* [M+H]+ in the TIC.
