# Supplementary material for: Glutathione reactivity with aliphatic polyisocyanates
Source: PLoS One. 2022 Jul 15;17(7):e0271471. doi: 10.1371/journal.pone.0271471 (PMC9286259; doi:10.1371/journal.pone.0271471)
Supplement: S7 Fig — The minor product of HDI biuret following control reaction in water without GSH or buffer possess characteristics consistent with the intramolecularly reacted product shown. (PDF) [file pone.0271471.s007.pdf]

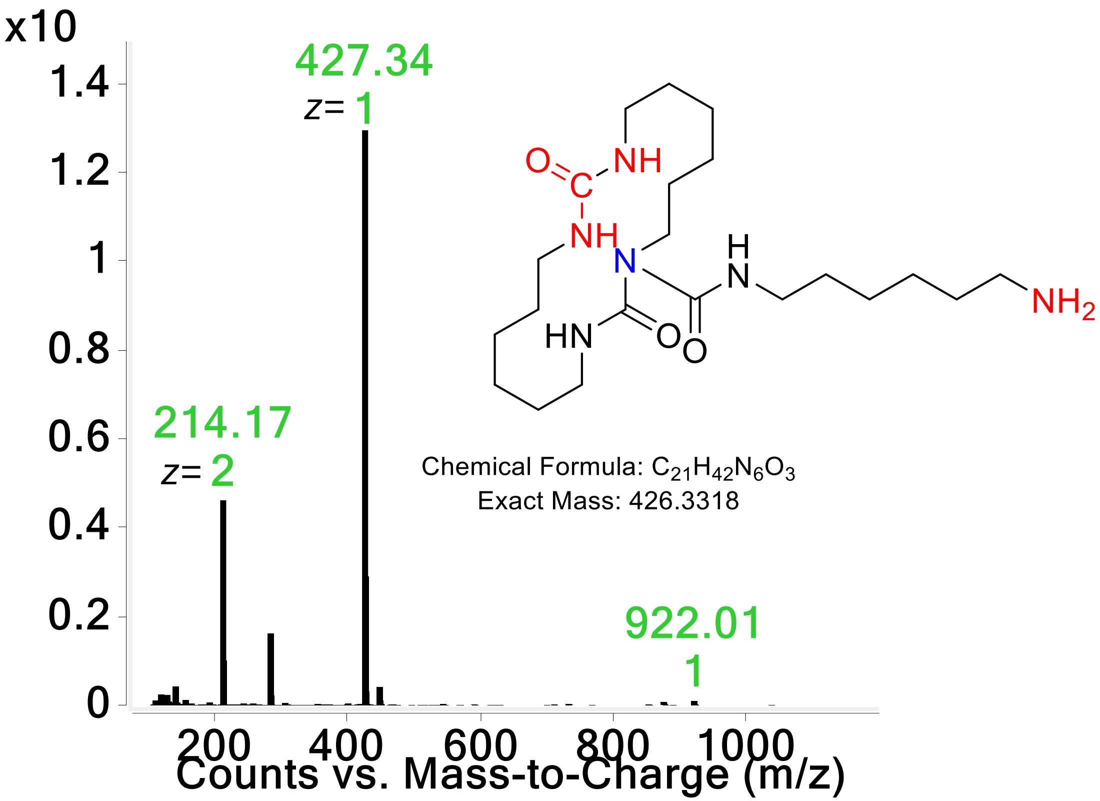

S7 Fig. LC-MS of dominant peak when HDI biuret is "reacted" in water (without buffer). The minor product of HDI biuret following control reaction in water without GSH or buffer possess characteristics consistent with the intramolecularly reacted product shown. Note 922.01 *m/z* [M+H]+ ion is internal reference standard.
