# Supplementary material for: Glutathione reactivity with aliphatic polyisocyanates
Source: PLoS One. 2022 Jul 15;17(7):e0271471. doi: 10.1371/journal.pone.0271471 (PMC9286259; doi:10.1371/journal.pone.0271471)
Supplement: S10 Fig — The [M+H]+ ions predicted to result from CID of the major reaction product of GSH with HDI uretdione under physiologic pH. (PDF) [file pone.0271471.s010.pdf]

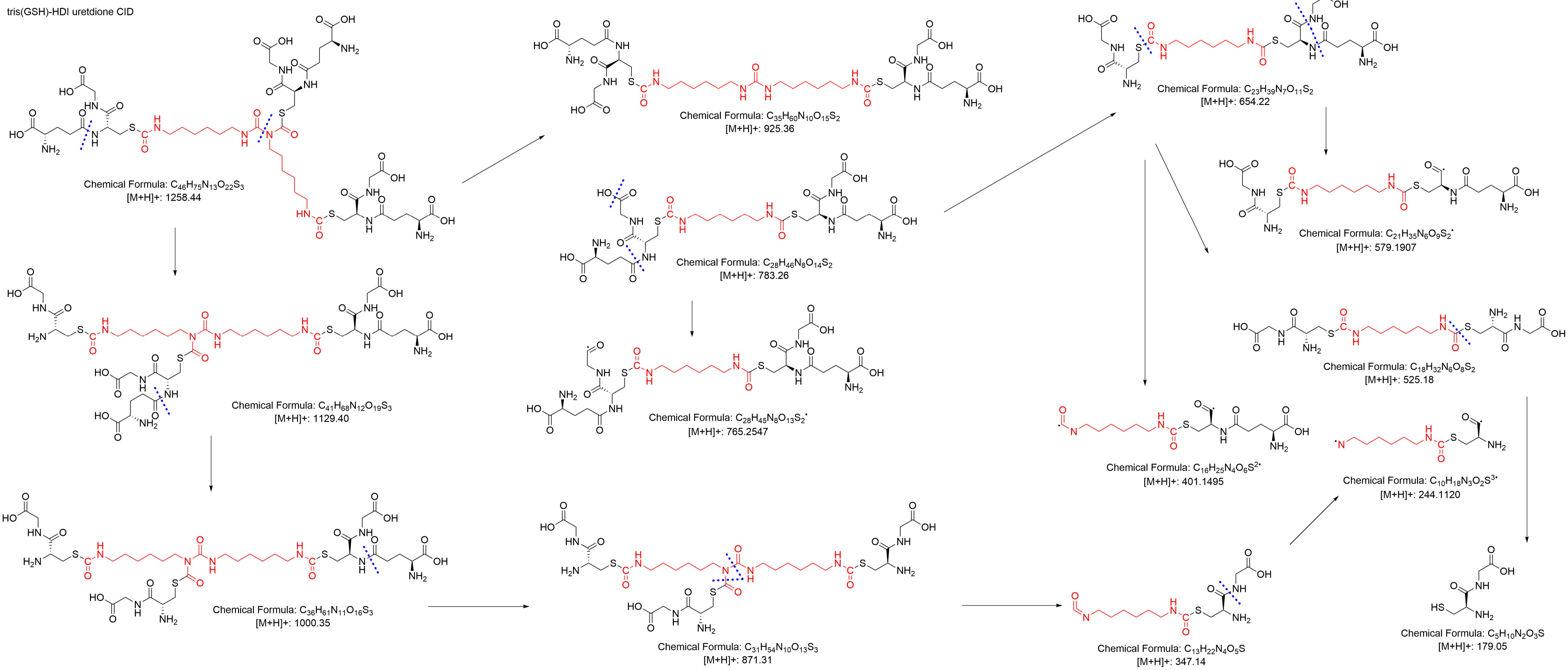

S10 Fig. Expected fragmentation pattern for tris(GSH)-HDI uretdione upon CID in MS/MS. The [M+H]+ ions predicted to result from CID of the major reaction product of GSH with HDI uretdione under physiologic pH.
