# Supplementary material for: Glutathione reactivity with aliphatic polyisocyanates
Source: PLoS One. 2022 Jul 15;17(7):e0271471. doi: 10.1371/journal.pone.0271471 (PMC9286259; doi:10.1371/journal.pone.0271471)
Supplement: S13 Fig — (A) Mass spec analysis of sample eluting from reverse phase LC column ~ 2.2 minutes. (B) the CID fragmentation spectra of the 618.29 m/z [M+H]+ ion upon MS/MS. (C) structural model for a second reaction product of GSH with HDI uretdione that occurs in the absence of pH buffer (i.e., pH < 4.0) based on exact mass and expected fragmentation pattern. (PDF) [file pone.0271471.s013.pdf]

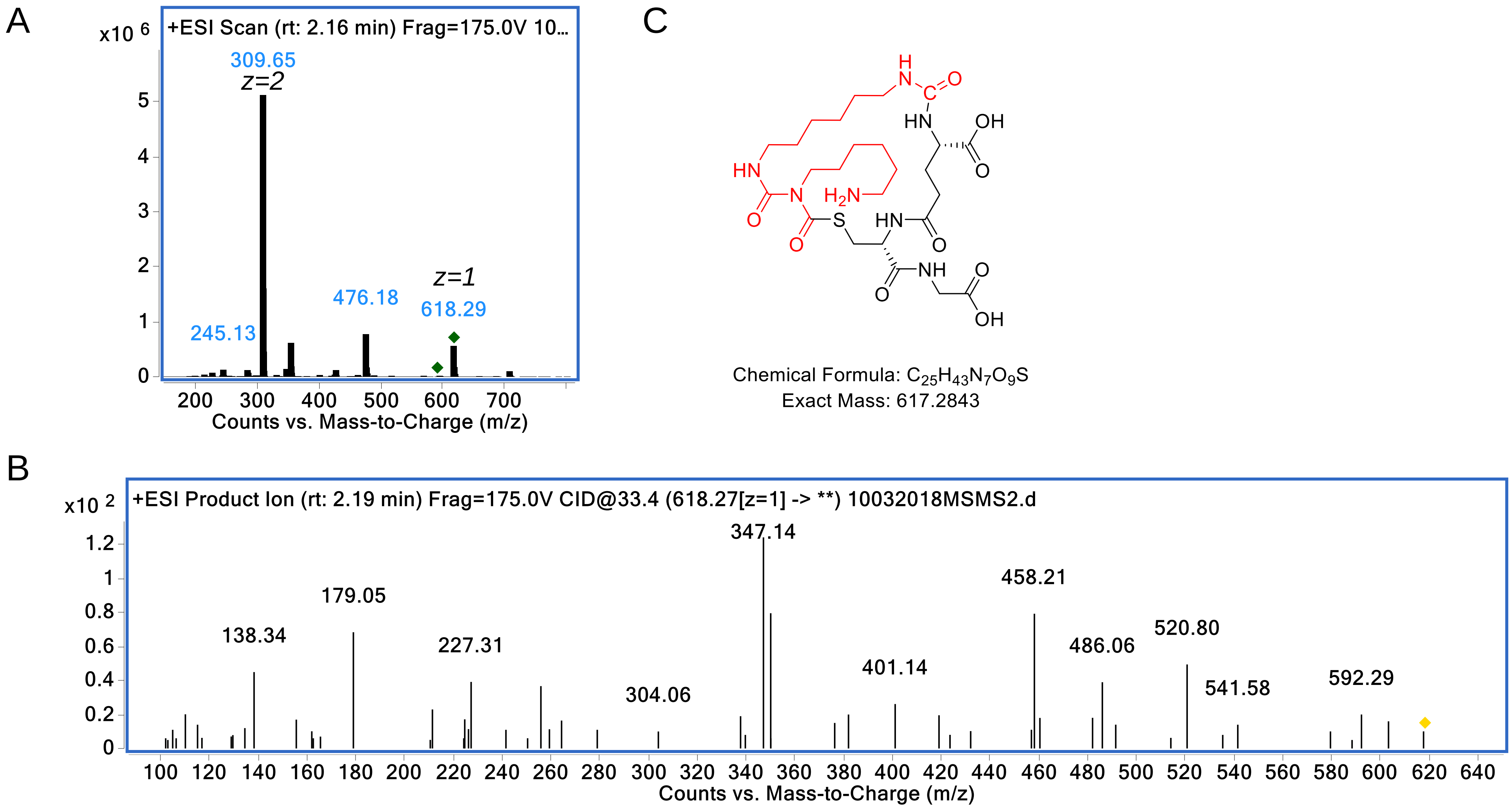

S13 Fig. Characterization of GSH reaction products with HDI uretdione in solution without pH buffer (i.e. pH < 4). (A) Mass spec analysis of sample eluting from reverse phase LC column ~ 2.2 minutes. (B) the CID fragmentation spectra of the 618.29 *m/z* [M+H]+ ion upon MS/MS. (C) structural model for a second reaction product of GSH with HDI uretdione that occurs in the absence of pH buffer (i.e. pH < 4.0) based on exact mass and expected fragmentation pattern (not shown).
