# Supplementary material for: Glutathione reactivity with aliphatic polyisocyanates
Source: PLoS One. 2022 Jul 15;17(7):e0271471. doi: 10.1371/journal.pone.0271471 (PMC9286259; doi:10.1371/journal.pone.0271471)
Supplement: S14 Fig — Human albumin was co-incubated with control (lanes 1, 3, 5) or GSH-HDI isocyanurate reaction products (lanes 2, 4, 6) overnight at 37°C, pH 9.0 followed by SDS-PAGE under reducing conditions and Coomassie blue staining or Western blotting with pooled serum IgG from unexposed individuals or HDI isocyanurate exposed workers as labeled. Note shift in electrophoretic migration of albumin following co-incubation with GSH-HDI isocyanurate consistent with conformational change that is recognized specifically by pooled serum IgG from exposed workers. (B) Right side shows hypothetical carbamylation of human albumin by GSH-isocyanurate reaction product, resulting in stable conjugation to lysine residues. Di-lysine motifs of albumin are preferred reaction sites for di-isocyanates, including “monomeric” HDI vapor [21, 27]. (PDF) [file pone.0271471.s014.pdf]

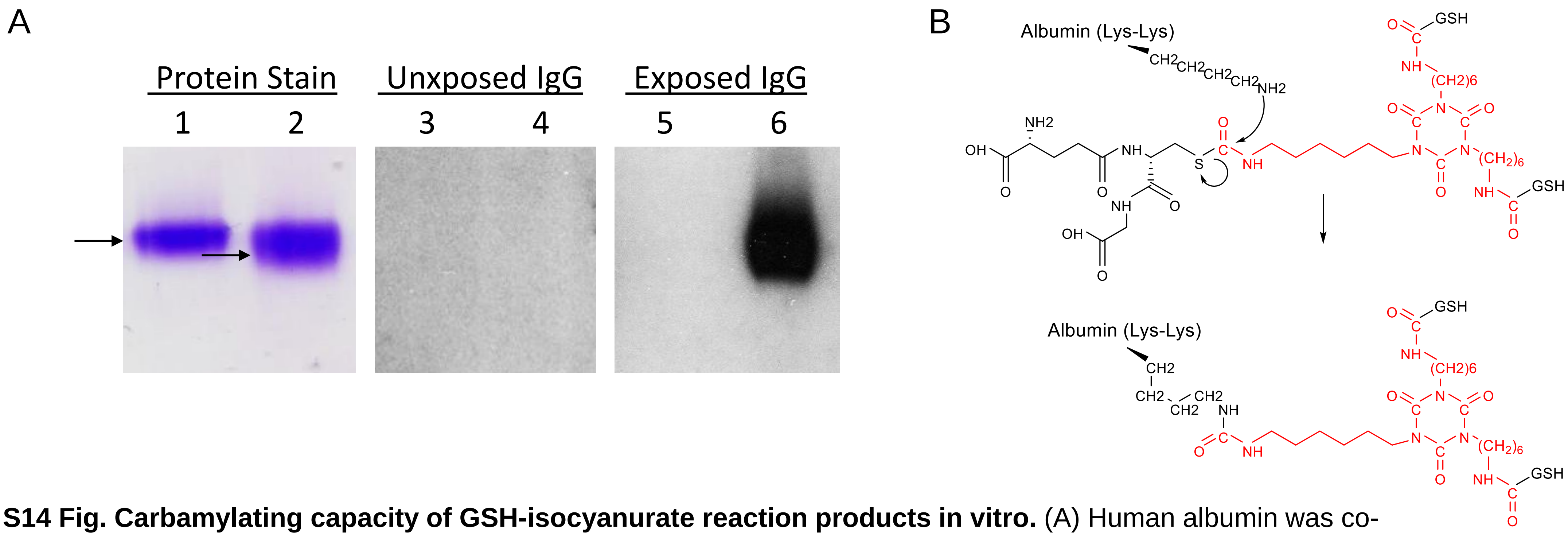


**S14 Fig. Carbamylating capacity of GSH-isocyanurate reaction products in vitro.** (A) Human albumin was co-incubated with control (lanes 1, 3, 5) or GSH-HDI isocyanurate reaction products (lanes 2, 4, 6) overnight at 37$^{o}$C, pH 9.0 followed by SDS-PAGE under reducing conditions and Coomassie blue staining or Western blotting with pooled serum IgG from unexposed individuals or HDI isocyanurate exposed workers as labeled. Note slight shift in electrophoretic migration of albumin following co-incubation with GSH-HDI isocyanurate consistent with conformational change that is recognized specifically by pooled serum IgG from exposed workers. Note, no binding was observed with polyclonal antiserum specific for HDI 'monomer" (not polymeric HDI). (B) Right side shows hypothetical carbamylation of human albumin by GSH-isocyanurate reaction product, resulting in stable conjugation to lysine residues. Di-lysine motifs of albumin are preferred reaction sites for di-isocyanates, including "monomeric" HDI vapor (see references 21 and 27 of manuscript).
