# Supplementary material for: Glutathione reactivity with aliphatic polyisocyanates
Source: PLoS One. 2022 Jul 15;17(7):e0271471. doi: 10.1371/journal.pone.0271471 (PMC9286259; doi:10.1371/journal.pone.0271471)
Supplement: S15 Fig — Bronchoalveolar lavage fluid from different mice given GSH-HDI uretdione reaction products (EU) or controls (C), once daily X 5 days, were western blotted with a mAb that specifically recognizes aliphatic isocyanate conjugated proteins (Panel A). The band at ~68 kDa likely reflects albumin, the best recognized “carrier” protein for diisocyanates in vivo and dominant airway fluid protein. Western blots were negative with polyclonal antibody that specifically recognizes HDI (monomer)-conjugated proteins (not shown) and control murine IgM (Panel B). *Note BAL fluid was depleted of immunoglobulin using protein G, mice were B-cell deficient, and anti-aliphatic isocyanate mAb is IgM isotype. (PDF) [file pone.0271471.s015.pdf]

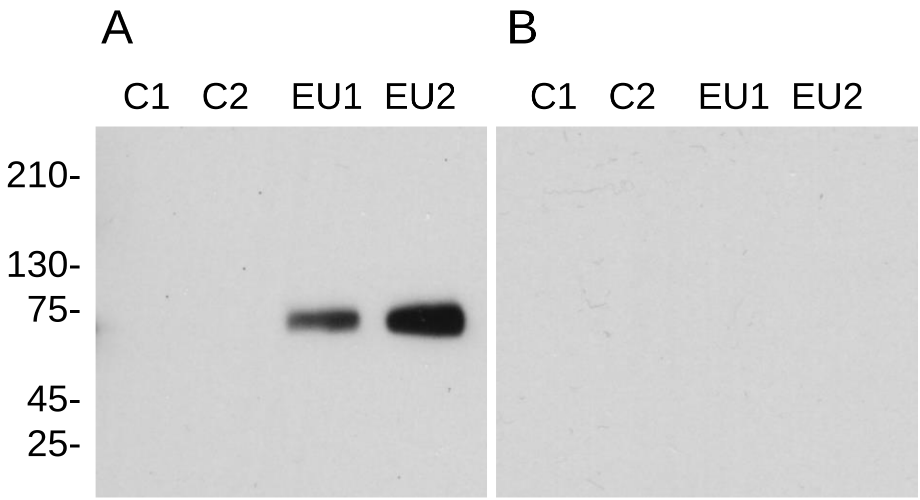

S15 Fig. Carbamylating capacity of GSH-HDI uretdione reaction products in vivo. Bronchoalveolar lavage fluid from different mice given GSH-HDI uretdione reaction products (EU) or controls (C), once daily X 5 days, were western blotted with a mAb that specifically recognizes aliphatic isocyanate conjugated proteins (Panel A). The band at ~68 kDa likely reflects albumin, the best recognized “carrier” protein for diisocyanates in vivo and dominant airway fluid protein. Western blots were negative with polyclonal antibody that specifically recognizes HDI (monomer)-conjugated proteins (not shown) and control murine IgM (Panel B). *Note BAL fluid was depleted of immunoglobulin using protein G, mice were B-cell deficient, and anti-aliphatic isocyanate mAb is IgM isotype.
